# Effects of Brain Wave Vibration Training on the Pain and Fatigue Disturbance Symptom Cluster in Persons with Cancer: A Randomized Controlled Trial

**DOI:** 10.3390/healthcare11070956

**Published:** 2023-03-27

**Authors:** Nam-Gi Lee, Byeong-Kwan Kim

**Affiliations:** 1 Department of Physical Therapy, Kwangju Women’s University, Gwangjuyeodae-gil 40, Gwangsan-gu, Gwangju 62396, Republic of Korea; 2 I Brain Development Center, Eungubi-ro 18, Yuseong-gu, Daejeon 34087, Republic of Korea

**Keywords:** brain wave vibration, cancer-related fatigue, cancer-related pain, mind–body interventions

## Abstract

Pain and fatigue disturbance constitute the most common symptom cluster in persons with cancer, causing physical and psychological problems associated with a decreased quality of life. This study investigated the effects of brain wave vibration (BWV) training on the pain and fatigue disturbance symptom cluster in persons with cancer. A total of 43 participants were voluntarily recruited and randomly assigned to the experimental group (BWV with therapeutic massage, n = 25) or the control group (conventional physical therapy with spontaneous physical activity, n = 18) (Randomized controlled trial registration number: KCT0005843). BWV with therapeutic massage was performed for a total of 24 sessions (70 min/day, 2 days/week for 12 weeks). The Breakthrough Pain Assessment Tool and Brief Fatigue Inventory were used to evaluate cancer-related pain (CRP) and cancer-related fatigue (CRF), respectively. Regarding CRP variables, the experimental group demonstrated significant improvements in the worst and typical episodes of breakthrough pain, pain distress, and interference with living a normal life compared to the control group. In CRF, there was no significant difference between the groups, although BWV training with therapeutic massage resulted in a significant difference between before and after the intervention. Therefore, our study suggests that BWV training with therapeutic massage is beneficial for improving CRP and CRF in cancer survivors.

## 1. Introduction

Pain and fatigue disturbance constitute the most common symptom cluster experienced by persons with cancer [1], causing physical and psychological problems associated with a decreased quality of life (QOL) [2]. Pain is defined as an unpleasant sensory or emotional experience related to actual or potential tissue damage or described in terms of such damage [3]. Cancer-related pain (CRP) refers to pain associated with cancer or its treatment [4]. CRP has somatosensory (e.g., suffering) and emotional (e.g., anxiety and depression) components and influences the activities of daily living and social participation of affected persons [5,6]. CRP, along with fatigue, is a major problem for cancer survivors and has a negative impact on QOL [7]. Cancer-related fatigue (CRF) is a persistent and subjective sense of physical, emotional, or cognitive tiredness related to cancer or its treatment that interferes with normal functioning [8]. In patients with advanced cancer, fatigue develops more rapidly and is longer-lasting and more severe than typical fatigue. CRF is a common adverse effect of cancer treatment, occurring in 30–80% of patients receiving radiotherapy [9]. Meanwhile, CRP has been reported in 59% of patients receiving anticancer therapy and in 64% of patients with advanced, metastatic, or terminal disease [10]. Traditionally, the management of CRP and CRF has focused on pharmaceutical treatments such as analgesics, psychostimulants, hematopoietic growth factors, and sedatives. Specific medications are prescribed for each symptom (pain or fatigue); however, they may have unintentional side effects. For example, opioid pain medications may cause a feeling of tiredness that can induce increased daytime sleepiness, eventually leading to sleep disturbance at night. Based on these clinical side effects, the pain–fatigue symptom cluster is difficult to manage using medications alone. Therefore, mind–body interventions are used as complementary therapy strategies, which are nonpharmaceutical, inexpensive, and noninvasive methods with relatively few side effects and may provide beneficial effects when added to traditional treatments [11]. Mind–body interventions focus on the interactions among the brain, mind, body, and behavior and consider the emotional, mental, social, spiritual, and behavioral factors that can directly affect health. These interventions include yoga, Pilates, meditation, massage therapy, relaxation techniques, Tai Chi/Qi Gong, healing touch, hypnotherapy, and brain wave vibration (BWV) [6]. BWV is an eclectic form of yoga and meditation known as “moving meditation.” It is designed to focus on bodily sensations (e.g., mechanoreception and proprioception) and to facilitate mind relaxation and the release of negative emotions through natural rhythmic movements [12]. In other words, BWV is based on the principle that gentle rhythmic shaking of one or more body parts generates vibrations that spread throughout the body, inducing a feeling of bodily relaxation and creating a peaceful, positive mind [13]. Such vibrations may strengthen the arousal systems of the brain, such as the brainstem reticular activating system, which is a core sleep–energy center and part of the subcortical–cortical arousal axis controlling energy distribution. This theory was developed in the 1950s and led to perennial arousal theories in psychophysiology [12,14]. In 2010, Jung et al., studied the effects of mind–body training on stress, positive affect, and plasma catecholamine (norepinephrine, epinephrine, and dopamine) levels and found that the BWV group (healthy participants who regularly practiced meditation) showed lower stress levels, greater positive affect, and higher plasma dopamine levels than the control group [15]. Kim et al., later suggested that BWV training could be used as a noninvasive intervention to improve anxiety, depression, fatigue, and QOL in women undergoing radiotherapy for breast cancer [16].

Although BWV training in patients with cancer has not been thoroughly investigated, several previous studies have reported its beneficial effects on improving perceived stress, positive affect, depression, sleep latency, and QOL in healthy persons [15,17]. Traditional meditation training has been shown to significantly reduce depression, anxiety, fatigue, and sleep disturbance and to improve the stress response, positive affect, and QOL in persons with breast cancer [18,19]. Studies on CRP management have revealed that physical exercise may impact the nociceptors with potential benefits in pain control, and on the other hand, higher levels of physical activity are related to lower pain levels [20]. Moreover, therapeutic massage has been shown to have positive effects on improving CRP and CRF in patients receiving chemotherapy; however, evidence for the analgesic effects of massage is still lacking [6,21]. To our knowledge, the effects of BWV training on pain reduction have not been previously investigated in patients with cancer. Therefore, we designed a BWV training program that includes physical exercise suitable for cancer patients and applied it together with therapeutic massage in this study. The purpose of the present study was to investigate the effects of the BWV training on the pain and fatigue disturbance symptom cluster in persons with cancer.

## 2. Materials and Methods

### 2.1. Participants

**Figure 1:**
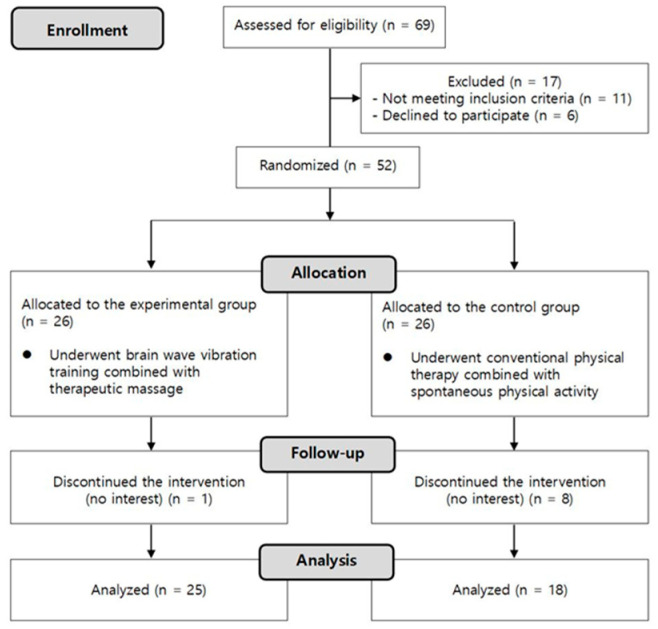
Flow diagram.

A total of 43 participants with a cancer diagnosis (33 women; age, 57.7 ± 8.5 years) were voluntarily recruited from a cancer rehabilitation center. This two-arm randomized controlled trial was conducted at this center from December 2020 to August 2021. The inclusion criteria for the participants were as follows: (1) age between 30 and 70 years, with a known cancer diagnosis; (2) currently undergoing surgery, chemotherapy, or radiotherapy; (3) able to communicate and participate in a survey; (4) have not been diagnosed with other psychological and neurological disorders; and (5) suffering from CRP and CRF, as assessed by simple pain and fatigue scales. The demographic and clinical characteristics of the participants are presented in Table 1. The experimental protocol was approved by the Institutional Review Board of the University of Brain Education (approval no. 202012-01), and written informed consent was obtained from all participants before study initiation. The randomized controlled trial (registration no. KCT0005843) was registered in the Clinical Research Information Service (CRiS) of the Republic of Korea, which is listed by the World Health Organization International Clinical Trials Registry Platform. Randomization to groups was performed using coin tossing [22]. The control and experimental groups consisted of 18 and 25 participants, respectively. Nine participants discontinued the intervention because of a lack of interest during the study (Figure 1). 

### 2.2. Intervention

Based on the randomized group assignments, 18 participants underwent standardized conventional physical therapy together with spontaneous physical activity (control group), and 25 participants underwent BWV training combined with therapeutic massage (experimental group). These interventions were performed after surgery and during the chemotherapy or radiation therapy period, 2 days/week for 12 weeks. In other words, the subjects received the intervention before or after the cancer treatment session because each subject’s chemotherapy or radiation therapy period was different. In the control group, conventional physical therapy (e.g., hot-pack treatment, ultrasound therapy, and electrical stimulation) was performed for a total of 24 sessions (50 min/day, 2 days/week for 12 weeks). Additionally, spontaneous physical activity was performed after conventional physical therapy for 20 min, with or without meditation. In the experimental group, therapeutic massage was performed by a trained and certified physical therapist before BWV (or moving meditation) training to increase circulation, improve muscle tissue metabolism and elasticity, and promote muscle relaxation. The massage was started with the participants lying prone. Gentle rhythmic gliding strokes, kneading, compression, vibration, tapping, and traction were applied to the neck, back, buttocks, and extremities. The therapeutic massage protocol was occasionally altered to avoid tumor or surgical sites and to adapt the depth of touch according to individual tolerance [21]. BWV training consisted of five steps: (1) warm-up; (2) stretching; (3) breathing control; (4) BWV combined with Ji-gam, meditation, and active movements; and (5) cool-down [15,17]. During the warm-up, patting exercises were performed to release stagnant energy, stimulate the “meridians” of the body, open acupressure points, and enhance energy/blood circulation and body awareness. Stretching was performed to open the meridians and promote energy circulation. Breathing control was performed in postures considered to accumulate energy in the abdomen (the “energy core”) and to lead to improved concentration, immunity, and health [12,13]. BWV was performed together with Ji-gam, meditation, and active movements. BWV training involves whole-body rhythmic movements that revitalize the body, improve mood, and enhance brain health. Ji-gam is a sitting energy meditation that is part of BWV meditation. It is designed to improve concentration and peace of mind. BWV meditation is performed by gentle rhythmic shaking of the head and neck while focusing on a positive thought. These actions result in vibrations that spread throughout the body, leading to relaxation of the body and a peaceful, positive mind [13]. Additionally, active movements comprised physical exercises suitable for patients with cancer, including upper- and lower-extremity movements (e.g., shoulder and hip circumduction motion, prone leg lifting, and toe–fingertip touch in the crossed long-sitting posture) and trunk movements (e.g., lumbar twisting motion). Finally, cool-down was performed by stretching. BWV combined with therapeutic massage was performed for a total of 24 sessions (70 min/day, 2 days/week for 12 weeks).

### 2.3. Measurements

#### 2.3.1. Breakthrough Pain Assessment Tool (BAT)

The BAT, developed by Weber et al., in the United Kingdom, consists of 14 items for assessing pain and current pain treatment. It includes questions for evaluating pain intensity and identifying interference with functioning (e.g., general activities, mood, walking, work, enjoyment of life, relations with others, and sleep). The pain intensity items evaluate current pain, worst pain, average pain, and least pain using an 11-point Numerical Rating Scale (NRS), with 0 indicating “no pain” and 10 indicating “pain as bad as you can imagine.” The NRS is also used for evaluating distress/side effects from painkillers (0 = “not at all” to 10 = “very much”) and the efficacy of painkillers (0 = “not at all effective” to 10 = “completely effective”). A categorical scale was used to identify the frequency and duration of breakthrough pain episodes. The causes of breakthrough pain and the type, dose, and side effects of painkillers were written in free text, and the pain locations were identified using a body-shaped image [23,24]. The reliability and validity of the BAT have been established in a sample of 100 cancer patients. The Cronbach’s alpha coefficient for reliability was 0.70 for all BAT items, 0.75 for the breakthrough pain severity and impact factor, and 0.55 for the breakthrough pain duration and medication effectiveness factor. The concurrent validity was high (*r* = 0.34–0.89), as demonstrated by high correlations with the Brief Pain Inventory in persons with cancer [24].

#### 2.3.2. Brief Fatigue Inventory (BFI)

The BFI comprises nine items asking patients whether they felt abnormally fatigued or tired during the last week [25]. It includes three questions for evaluating fatigue and six questions for identifying fatigue-related interferences (e.g., general activities, mood, walking, normal work, relations with other people, and enjoyment of life). The fatigue measures assess the patients’ worst fatigue, average fatigue, and fatigue during the previous 24 h using an 11-point NRS, with 0 indicating “no fatigue” and 10 indicating “fatigue as bad as you can imagine.” The fatigue-related interference scores range from 0 (“does not interfere”) to 10 (“completely interferes”). The total BFI score was calculated as the mean value of all nine items [26,27]. The reliability of the BFI-Korean version (BFI-K) has been established, with Cronbach’s alpha coefficients of 0.956 and 0.955 in cancer patients and controls, respectively. For the concurrent validity of the BFI-K, the total score and individual scores of all nine items showed excellent correlations (Pearson’s correlation coefficient, *r* = 0.50–0.66; *p* < 0.0001) with the fatigue sub-item of the European Organization for Research and Treatment of Cancer QLQ-C30 in cancer patients [26].

### 2.4. Statistical Analysis

Descriptive statistics included median values, interquartile ranges (IQRs), and means and standard deviations (SDs). The Kolmogorov–Smirnov test was used to assess the normality of the variables. Categorical variables are presented as sums and percentages. The open-ended questions in the BAT were converted to categorical variables. Continuous variables are presented as medians and IQRs or as means and SDs, and significant differences within each group and between groups (experimental and control groups) were analyzed according to the nature of each variable. For nonparametric statistics, a Wilcoxon matched-pair signed-rank test was used to assess significant within-group differences, and the Mann–Whitney U test was used to evaluate significant between-group differences using change values between pre- and post-tests. For parametric statistics, paired and independent *t*-tests were used to analyze the data. All statistical analyses were performed using SPSS for Windows (version 21.0; SPSS Inc., Chicago, IL, USA). The level of significance was set at 0.05.

## 3. Results

The locations of pain are presented in Table 2. The lower extremities (n = 12 (48.0%) in the pre- and post-tests), waist (n = 8 (32.0%) in the pre-test and n = 7 (28.0%) in the post-test), shoulder (n = 8 (32.0%) in the pre-test and n = 5 (20.0%) in the post-test), upper extremities (n = 7 (28.0%) in the pre-test and n = 4 (16.0%) in the post-test), and neck (n = 6 (24.0%) in the pre-test and n = 3 (12.0%) in the post-test) were the most common pain locations in the experimental group. In the control group, the most common pain locations were the lower extremities (n = 8 (44.4%) in the pre-test and n = 7 (38.9%) in the post-test), upper extremities (n = 5 (27.8%) in the pre-test and n = 2 (11.1%) in the post-test), and waist (n = 3 (16.7%) in the pre-test and n = 4 (22.2%) in the post-test).

The pain frequency, trigger factors, and pain-relief factors are shown in Table 3. With respect to the frequency of pain, ”more than 4 times a day” was selected by most of the participants in the experimental (n = 9 (36.0%) in the pre-test and n = 5 (20.0%) in the post-test) and control (n = 6 (33.3%) in the pre- and post-tests) groups. The trigger factors of pain, including surgery, chemotherapy, radiotherapy, and maintenance of the sitting posture, were reported as existent by 16 (64.0%) experimental group participants in both the pre- and post-tests and by 9 (50.0%) and 7 (38.9%) control group participants in the pre- and post-tests, respectively. Pain-relief factors, including the use of painkillers, manual therapy, meditation, and stretching exercises, were reported as existent by 14 (56.0%) and 17 (68.0%) experimental group participants in the pre- and post-tests, respectively, and by 13 (72.2%) and 10 (55.6%) control group participants in the pre- and post-tests, respectively.

The use of painkillers and their effects are presented in Table 4. According to the pre-test and post-test (n = 19, 76.0%) data, many experimental group participants did not use painkillers. Similarly, many control group participants reported not using painkillers in the pre-test (n = 8, 44.4%) and post-test (n = 11, 61.1%). With respect to the meaningful effects of painkillers, most of the participants selected ”> 30 min” in both the experimental (n = 3 (12.0%) in the pre-test and n = 5 (20.0%) in the post-test) and control (n = 8 (44.4%) in the pre-test and n = 5 (27.8%) in the post-test) groups. A few participants (n = 1 (4.0%) in the experimental group; n = 1 (5.6%) in the control group) reported side effects such as dizziness and headache in both the pre- and post-tests.

The results on pain characteristics (lasting time and worst and typical episodes), pain distress, and interference with normal living are shown in Table 5. In the within-group comparisons, all variables revealed significant differences (*p* = 0.000 to *p* = 0.003) between the pre- and post-tests in the experimental group, whereas the control group showed no significant differences (*p* = 0.256 to *p* = 0.859). In the between-group comparisons, the worst and typical pain episodes, pain distress, and interference with normal living showed significant differences (*p* = 0.012, *p* = 0.000, *p* = 0.006, and *p* = 0.019, respectively).

The fatigue results are presented in Table 6. In the within-group comparison, fatigue showed a significant difference (*p* = 0.000) in the experimental group but not in the control group (*p* = 0.462). Although no significant difference was found between the groups, the difference between interventions showed a trend toward significance (*p* = 0.070).

## 4. Discussion

Although studies have been published on the effects of the BWV (moving meditation) training on perceived stress, fatigue, positive affect, depression, sleep latency, and QOL in healthy people [15,17], few studies have been conducted on the pain and fatigue disturbance symptom cluster that is commonly experienced by persons with cancer. This is the first study to investigate the effects of BWV training on the pain–fatigue symptom cluster in persons with cancer. The main findings of our study were as follows: (1) for CRP, BWV training with therapeutic massage demonstrated significant improvements in the worst and typical pain, pain distress, and interference with normal living compared with combined conventional physical therapy and spontaneous physical activity; (2) for CRF, no significant difference between groups was found, although BWV training with therapeutic massage showed a significant difference between before and after the intervention. Thus, this study contributes to the development of complementary therapy strategies for CRP and CRF management in persons with cancer.

In terms of CRP, our results demonstrated that BWV training with therapeutic massage (the experimental intervention) provided significant improvements in the lasting time of pain, worst and typical pain, pain distress, and interference with normal living, whereas the control intervention showed no significant enhancements. Our results are difficult to compare with those of previous studies because this is the first study to examine the effects of BWV training on pain in persons with cancer. Mind–body therapies, such as Pilates and yoga, have been investigated to determine whether they have significant effects on pain variables in persons with cancer [28]. Alpozgen et al. (2016) investigated the effects of Pilates-based exercises (PE) in comparison with combined passive and active exercises (CE) and home exercises (HE) in patients with upper-extremity disorders related to breast cancer treatment. They observed that pain, range of motion (ROM), and functional status showed significant improvements after the PE intervention (pain intensity: *p* < 0.001 (motion), *p* = 0.004 (rest); ROM: *p* = 0.001 to *p* = 0.017; functional status: *p* < 0.001); CE and HE also resulted in meaningful changes in these variables [29]. Danhauer et al. (2021) examined the effects of a mindful yoga program on pain reduction in women with metastatic breast cancer and suggested that the program was associated with acute improvements (*p* < 0.001) in CRP [30]. In a literature review on cancer rehabilitation for chronic pain management, the positive effect of mind–body therapies such as Pilates [31] and yoga [30] in patients with breast cancer was more prominent for symptoms related to psychological well-being, including anxiety, stress, and depression [28,32]. Bower et al. (2015) also found that mindfulness meditation led to a decrease in perceived stress, depression, inflammatory activity (e.g., pro-inflammatory gene expression and inflammatory signaling), fatigue, and sleep disturbance and an increase in positive mood in younger breast cancer survivors [18]. Similarly, massage therapy was also related to a reduction in anxiety and stress and may provide relief from physical symptoms such as pain by enhancing the sense of personal well-being [32,33].

In terms of CRF, our results demonstrated that BWV training with therapeutic massage resulted in a meaningful change in fatigue variables between before and after the intervention, whereas the control intervention provided no significant improvements. This result is consistent with one previous study [16]. Kim et al. (2013) examined the effects of BWV meditation on anxiety, depression, fatigue, and QOL in 102 women undergoing radiotherapy for breast cancer and compared the effects of radiotherapy with BWV meditation therapy and conventional radiotherapy alone. They found that breast cancer patients who underwent BWV meditation therapy showed significant improvements in anxiety (*p* = 0.032), fatigue (*p* = 0.030), and global QOL (*p* = 0.028) [16]. Although few studies have been published on BWV intervention, several studies have reported on mind–body interventions such as progressive muscle relaxation, mindfulness meditation, yoga, and Qi Gong/Tai Chi Easy (QG/TCE) [19,34,35]. Metin et al. (2019) reported that progressive muscle relaxation and mindfulness meditation resulted in a significant reduction in BFI scores compared to a single-time attention-matched education in patients with early breast cancer [19]. In a randomized controlled trial study, Lin et al. (2019) investigated the influence of a yoga therapy program on CRF and the mediational relationship between changes in sleep and CRF in 410 cancer survivors. They found that the 4-week yoga therapy program resulted in significantly superior improvements in CRF (*p* < 0.01) compared to a standard care program. They suggested that the improvements in CRF (22–37%) from the yoga therapy program were associated with improvements in sleep quality and daytime functioning [34]. Another study demonstrated that an 8-week yoga therapy program significantly reduced CRF (general fatigue: *p* = 0.033 and physical fatigue: *p* = 0.048) and depression (*p* < 0.001) in 173 cancer patients experiencing mild to severe fatigue. Zetzl et al. (2021) suggested that yoga therapy is useful for reducing CRF (particularly the physical aspects of fatigue) in women with breast cancer [35]. In another study, the investigators compared the effects of QG/TCE (or meditative movement practice) on fatigue, depression, and sleep quality with those of a sham intervention. They observed significant improvements in fatigue after the intervention (*p* = 0.005) and at the 3-month follow-up (*p* = 0.024) in the QG/TCE group; however, there were no significant improvements in depression and sleep quality [36]. According to these previous studies, mind–body interventions influence CRP, CRF, and psychological well-being in persons with cancer. Moreover, several investigators have suggested that mind–body interventions, including meditation practice, Pilates, and yoga, are complementary therapies that may play an important role in cancer rehabilitation. Fraser et al. (2009) reported a hypogonadism prevalence of 75% in male cancer survivors receiving oral opioids for chronic pain management [37], and another study showed that 18 of 20 male cancer survivors receiving high-dose opioids had a lower total testosterone level than those who were not [38]. According to previous studies, opioid pain medications play an important role in the pathogenesis of hypogonadism in cancer survivors. Hypogonadism also affects fatigue in persons with cancer. Greenfield et al. (2010) found that cancer survivors with hypogonadism had lower scores in the Functional Assessment of Chronic Illness Therapy-Fatigue (FACIT-F) questionnaire, indicating increased fatigue during their daily activities [39]. Therefore, our findings suggest that BWV training with therapeutic massage is a beneficial intervention for improving CRP and CRF and may provide additional effects when used in combination with traditional treatments in cancer survivors, including those receiving oral opioid medication therapy or chemotherapy.

The present study revealed significant findings regarding CRP and CRF. However, a few limitations were identified. First, although randomization was performed, there were differences in the baseline (or pretest) data between the two groups. In the control group, 8 of 26 cancer survivors with severe pain and fatigue did not feel the treatment effect and did not participate until the end of the intervention period. We suggest that the loss of these data might affect the difference in baseline data. Second, this study was not sufficiently blinded because only two well-trained examiners performed the interventions and data collection. Finally, the study participants had different types of cancer, including breast, gynecologic, colorectal, and lung cancers. Hence, further studies with a double-blind design and a large sample size are required to generalize the data according to cancer type. Additionally, further studies are needed to confirm the efficacy and possible scientific mechanisms of BWV training with therapeutic massage, such as the theoretical changes in brain activation areas or physiological variables resulting from the intervention. Notwithstanding these limitations, improvements in CRP and CRF were observed with combined BWV training and therapeutic massage, thereby supporting its therapeutic effectiveness.

## 5. Conclusions

CRP and CRF constitute the most common symptom cluster in cancer survivors, leading to physical and psychological problems related to decreased QOL. The traditional medical management of CRP and CRF focuses on pharmaceutical treatments; however, some medications have side effects. Mind–body intervention is a nonpharmaceutical, inexpensive, and noninvasive complementary therapy strategy. The present study investigated the effects of BWV training on the pain–fatigue symptom cluster in persons with cancer. BWV training with therapeutic massage provided significant improvements in both CRP variables, including the worst and typical pain, pain distress, and interference with normal living, and CRF variables. Therefore, our study suggests that BWV training with therapeutic massage is a helpful intervention for improving CRP and CRF and may provide additional effects when used in combination with traditional treatments in cancer survivors.

## Figures and Tables

**Table 1:** Demographic and clinical characteristics of participants (N = 43).

Variables	Categories	Experimental Group (n = 25)	Control Group (n = 18)
Age (years)		58.1 ± 5.6 ^a^	56.6 ± 9.9
Height (cm)		160.2 ± 8.2	161.3 ± 8.5
Weight (kg)		59.6 ± 7.5	58.6 ± 9.2
Gender	Male	6 (24.0) ^b^	4 (22.2)
	Female	19 (76.0)	14 (77.8)
Cancer type ^c^	Breast cancer	10 (40.0)	4 (22.2)
	Gynecologic cancer	5 (20.0)	3 (16.7)
	Colorectal cancer	4 (16.0)	4 (22.2)
	Lung cancer	5 (20.0)	3 (16.7)
	Gastric cancer	1 (4.0)	1 (5.6)
	Other cancers	3 (12.0)	3 (16.7)
Treatment ^d^	Surgery	22 (88.0)	12 (66.7)
	Chemotherapy	20 (80.0)	13 (72.2)
	Radiation therapy	9 (36.0)	4 (22.2)

Data are presented as the mean ± standard deviation ^a^ and n (%)^b^; ^c^ Some patients had more than one type of cancer; ^d^ Some patients received more than one treatment.

**Table 2:** Locations of pain.

Locations	Experimental Group (n = 25)		Control Group (n = 18)	
	Pre-Test	Post-Test	Pre-Test	Post-Test
Waist	8 (32.0) ^a^	7 (28.0)	3 (16.7)	4 (22.2)
Back	1 (4.0)	0 (0)	3 (16.7)	0 (0)
Neck	6 (24.0)	3 (12.0)	0 (0)	2 (11.1)
Pelvis	2 (8.0)	1 (4.0)	0 (0)	0 (0)
Lower extremities	12 (48.0)	12 (48.0)	8 (44.4)	7 (38.9)
Shoulder	8 (32.0)	5 (20.0)	1 (5.6)	3 (16.7)
Upper extremities	7 (28.0)	4 (16.0)	5 (27.8)	2 (11.1)
Abdomen	3 (12.0)	1 (4.0)	2 (11.1)	3 (16.7)
Elsewhere (e.g., chest, anus, surgical site)	4 (16.0)	5 (20.0)	5 (27.8)	5 (27.8)

^a^ Data are presented as n (%); Experimental group: brain wave vibration training with therapeutic massage; Control group: conventional physical therapy with spontaneous physical activity; Some participants selected multiple pain locations in the self-report questionnaire.

**Table 3:** Pain frequency, trigger factors, and pain-relief factors.

Variables	Categories	Experimental Group (n = 25)		Control Group (n = 18)	
		Pre-Test	Post-Test	Pre-Test	Post-Test
Pain frequency	Less than once a day	4 (16.0) ^a^	6 (24.0)	3 (16.7)	7 (38.9)
	1–2 times a day	3 (12.0)	8 (32.0)	5 (27.8)	3 (16.7)
	3–4 times a day	9 (36.0)	4 (16.0)	4 (22.2)	2 (11.1)
	More than 4 times a day	9 (36.0)	5 (20.0)	6 (33.3)	6 (33.3)
	Do not know	0 (0)	2 (8.0)	0 (0)	0 (0)
Trigger factors	Nonexistent	9 (36.0)	9 (36.0)	9 (50.0)	11 (61.1)
	Existent	16 (64.0)	16 (64.0)	9 (50.0)	7 (38.9)
Pain-relief factors	Nonexistent	11 (44.0)	8 (32.0)	5 (27.8)	8 (44.4)
	Existent	14 (56.0)	17 (68.0)	13 (72.2)	10 (55.6)

^a^ Data are presented as n (%); Experimental group: brain wave vibration training with therapeutic massage; Control group: conventional physical therapy with spontaneous physical activity; Trigger factors of pain included surgery, chemotherapy, radiotherapy, and maintenance of the sitting posture, whereas pain-relief factors included the use of painkillers, manual therapy, meditation, and stretching exercises.

**Table 4:** Use and effects of painkillers.

Variables	Categories	Experimental Group (n = 25)		Control Group (n = 18)	
		Pre-Test	Post-Test	Pre-Test	Post-Test
Use of painkillers	Non-use	19 (76.0) ^a^	19 (76.0)	8 (44.4)	11 (61.1)
	Use	6 (24.0)	6 (24.0)	10 (55.6)	7 (38.9)
Meaningful effects	No effect	0 (0)	0 (0)	0 (0)	0 (0)
	<10 min	1 (4.0)	0 (0)	0 (0)	0 (0)
	10–20 min	0 (0)	1 (4.0)	1 (5.6)	0 (0)
	20–30 min	2 (8.0)	0 (0)	1 (5.6)	2 (11.1)
	>30 min	3 (12.0)	5 (20.0)	8 (44.4)	5 (27.8)
Side effects	Existent	1 (4.0)	1 (4.0)	1 (5.6)	1 (5.6)

^a^ Data are presented as n (%); Experimental group: brain wave vibration training with therapeutic massage; Control group: conventional physical therapy with spontaneous physical activity; All participants knew the brand names of the painkillers but did not know their types; Side effects included dizziness and headache.

**Table 5:** Pain characteristics, pain distress, and interference with normal living.

Variables	Experimental Group (n = 25)		Within Group		Control Group (n = 18)		Within Group		Between Groups	
	Pre-Test	Post-Test	z	*p*	Pre-Test	Post-Test	z	*p*	z	*p*
Lasting time ^b^	3.0 (1.5–4.0) ^a^	2.0 (1.0–2.5)	−2.931	0.003 *	2.5 (1.0–4.0)	1.5 (1.0–4.0)	−0.358	0.720	−0.357	0.721
Worst episode	8.0 (7.0–8.0)	3.0 (1.5–5.0)	−3.981	0.000 *	3.5 (2.8–6.0)	5.0 (1.8–6.5)	−1.135	0.256	−2.500	0.012 ^†^
Typical episode	7.0 (5.0–8.0)	2.0 (1.0–3.5)	−4.241	0.000 *	3.0 (2.0–4.3)	3.0 (2.0–4.3)	−0.178	0.859	−3.709	0.000 ^†^
Pain distress	8.0 (5.0–9.0)	2.0 (1.0–5.0)	−4.025	0.000 *	3.0 (1.8–5.0)	3.0 (1.0–5.3)	−0.963	0.336	−2.732	0.006 ^†^
Interference with normal living	8.0 (6.0–9.0)	3.0 (1.0–5.0)	−3.744	0.000 *	3.0 (0.0–6.3)	2.0 (0.8–6.0)	−0.826	0.409	−2.351	0.019 ^†^

^a^ Data are presented as the median (interquartile range). ^b^ Lasting time: 1 = < 5 min, 2 = 5–15 min, 3 = 15–30 min, 4 = 30–60 min, 5 = > 60 min; Experimental group: brain wave vibration training with therapeutic massage; Control group: conventional physical therapy with spontaneous physical activity; * Significant difference (*p* < 0.05) within each group according to the Wilcoxon matched-pair signed-rank test; ^†^ Significant difference (*p* < 0.05) between groups according to the Mann–Whitney U test.

**Table 6:** Fatigue.

Variable	Experimental Group (n = 25)		Within Group		Control Group (n = 18)		Within Group		Between Groups	
	Pre-Test	Post-Test	t	*p*	Pre-Test	Post-Test	t	*p*	t	*p*
Fatigue	49.1 ± 23.0 ^a^	29.2 ± 18.4	4.557	0.000 *	30.9 ± 19.1	33.9 ± 22.9	−0.753	0.462	2.368	0.070

^a^ Data are presented as the mean ± standard deviation; Experimental group: brain wave vibration training with therapeutic massage; Control group: conventional physical therapy with spontaneous physical activity; * Significant difference (*p* < 0.05) within each group according to a paired *t*-test; No significant difference (*p* > 0.05) between groups according to an independent *t*-test.

## Data Availability

Not applicable.
